# Osteo‐F, a Newly Developed Herbal Formula, Ameliorates Osteoarthritis Through the NF‐κB/IκB/JNK Pathway Based on Network Pharmacology

**DOI:** 10.1002/fsn3.70239

**Published:** 2025-05-11

**Authors:** Seong Chul Jin, You Yeon Choi, Minwoo Song, Hee Kyung Baek, Seungyob Yi, Eun‐Jung Kim, Woong Mo Yang

**Affiliations:** ^1^ Department of Convergence Korean Medical Science, College of Korean Medicine Kyung Hee University Seoul Republic of Korea; ^2^ KHU‐KIST Department of Converging Science and Technology College of Korean Medicine, Graduate School, Kyung Hee University Seoul Republic of Korea; ^3^ Department of Acupuncture and Moxibustion, Graduate School of Oriental Medicine Dongguk University Goyang‐si Gyeonggi‐do Korea; ^4^ Korean Medicine Digital Convergence Center (KMDC) Kyung Hee University Seoul Republic of Korea

**Keywords:** functional foods, inflammation, network pharmacology, NF‐κB/IκB/JNK pathway, osteoarthritis

## Abstract

Osteoarthritis (OA) is a painful joint condition primarily caused by cartilage degradation, leading to pain and reduced mobility. Given the side effects of current treatments, this study investigates Osteo‐F, a novel herbal‐based functional ingredient formulated with *Schizandra chinensis*, *Lycium chinense* , and *Eucommia ulmoides* , traditionally valued for their bioactive and health‐promoting properties. Network pharmacology analysis identified significant interactions involving Osteo‐F within the TNF signaling pathway, highlighting its role in modulating key inflammatory processes in OA. In vivo experiments using a monosodium iodoacetate‐induced OA rat model demonstrated significant improvements in arthritis scores, bone mineral content, and bone mineral density, alongside preservation of cartilage integrity, as confirmed by histological analyses. In vitro studies further revealed that this formulation reduced the activation of JNK and NF‐κB pathways, decreasing inflammatory cytokines and matrix metalloproteinases critical in cartilage breakdown. These findings underscore the potential of Osteo‐F as a functional food candidate to reduce inflammation and support cartilage preservation in OA. Future clinical trials are required to validate these findings and explore its dietary integration in OA management.

## Graphical abstract

Osteo‐F, a newly developed herbal formula comprising *Schisandra chinensis*, *Lycium chinense* , and *Eucommia ulmoides* , was evaluated for its therapeutic effects in osteoarthritis (OA). Through network pharmacology, in vivo, and in vitro analyses, Osteo‐F was shown to regulate NF‐κB/IκB/JNK signaling and reduce inflammation, cartilage degradation, and bone loss. These findings suggest its potential as a functional food‐based strategy for OA management.

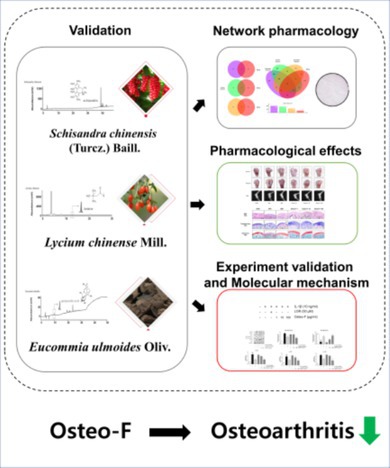

**Abbreviations**
BMC: bone mineral content
BMD: bone mineral density
c‐Jun: c‐Jun N‐terminal kinase
COL‐II: type II collagen
DXA: dual energy X‐ray absorptiometry
H&E: hematoxylin and eosin
IL: interleukin
IκB: inhibitor of kappa B
JNK: c‐Jun N‐terminal kinase
MIA: monosodium iodoacetate
MMP: matrix metalloproteinase
NF‐κB: nuclear factor kappa‐light‐chain‐enhancer of activated B cells
NSAIDs: nonsteroidal anti‐inflammatory drugs
OA: osteoarthritis
RT‐PCR: reverse transcription polymerase chain reaction

## 1 Introduction

Osteoarthritis (OA) is one of the most prevalent and debilitating joint conditions affecting individuals worldwide, characterized by the gradual degradation of cartilage (Kim et al. 2018). This results in pain, inflammation, and a reduced range of motion, significantly hampering day‐to‐day activities and turning mundane tasks into challenges. The risk of developing OA increases with age, highlighting its significance as a global health concern in an aging population (Loeser 2017).

Conventional treatments for OA, such as nonsteroidal anti‐inflammatory drugs and corticosteroids, have been the mainstay in symptom alleviation. However, these synthetic drugs, while effective, often come with a variety of side effects like stomach ulcers, liver damage, and an increased risk of heart disease (Bindu et al. 2020). Additionally, with the progression of OA, many conventional non‐invasive strategies often fall short of providing adequate relief. Recognizing these challenges, there is a growing interest in and emphasis on natural alternatives.

The Osteo‐F used in this study is a novel combination of three drugs, a unique blend of herbal medicine such as *Schisandra chinensis* (Turcz.) Baill ( *S. chinensis* ), *Lycium chinense* Mill. (*L. chinese*), and *Eucommia ulmoides* Oliv. ( *E. ulmoides* ). Osteo‐F has been approved by the Korean Ministry of Food and Drug Safety as an individually recognized ingredient for maintaining healthy bones (Recognition Number: 2024‐13, Approval Date: 2024.04.19). This approval covers an extract complex of *S. chinensis* , *L. chinense* , and *E. ulmoides* with a recommended daily intake amount of 253 mg/day, provided by Boin Bioconversion Co. Ltd. Our initial research into Osteo‐F confirmed its effectiveness in targeting osteoporosis by reducing bone loss and improving bone strength (Lee et al. 2017). Although osteoporosis and osteoarthritis are different diseases, they share common risk factors, and the roles of genetics and epigenetics in both conditions are related. Additionally, the osteoporotic, bone‐forming, and erosive subtypes observed in osteoporosis have also been identified in OA (Geusens and van den Bergh 2016). Building on the established benefits of these herbs, the promising results prompted us to hypothesize the possible efficacy of Osteo‐F in addressing the multifaceted challenges of OA. Specifically, *S. chinensis* has been used in traditional remedies to relieve joint pain and reduce inflammation (Panossian and Wikman 2008). Its lignans contribute to anti‐inflammatory and analgesic effects, supporting musculoskeletal health. Similarly, *L. chinense* has been applied in traditional medicine to improve joint function and alleviate rheumatic pains, nourishing the liver and kidneys, which are considered vital for bone and joint health in traditional Chinese medicine (Potterat 2010). *E. ulmoides* has been traditionally used to strengthen bones and muscles and to alleviate joint pain and stiffness associated with osteoarthritis (He et al. 2014). Its iridoids and lignans contribute to anti‐inflammatory and analgesic effects, directly impacting musculoskeletal health. These traditional uses suggest a potential therapeutic role for these herbs in treating OA.

In the present study, we employed a multi‐faceted approach to comprehensively assess the potential of Osteo‐F in treating OA. Initiating our investigation with advanced in silico analyses, we utilized protein network analysis to predict the potential molecular interactions and signaling pathways involved in its therapeutic action against OA. We followed this computational prediction with in vivo experiments to validate the observed effects.

## 2 Materials and Methods

### 2.1 Preparation of Osteo‐F

The fruits of *Schisandra chinensis* (Turcz.) Baill. ( *S. chinensis* ) and *Lycium chinense* Mill. ( *L. chinense* ), along with the radix of *Eucommia ulmoides* Oliv. ( *E. ulmoides* ), were sourced from the Munkyeong Omija Valley Agricultural Association (Munkeong, Korea). Voucher specimens were prepared for each plant material and archived at BOINBIO Convergence Co. Ltd., Seoulbioherb, Hoegi‐ro, Dongdaemun‐gu, Seoul, Republic of Korea, under the codes SC–B20001‐1 ( *S. chinensis* ), LC‐B20001‐1 ( *L. chinense* ), and EU‐B20001‐1 ( *E. ulmoides* ), respectively. To produce Osteo‐F, 100 kg of *S. chinensis* fruits underwent grinding to achieve a 20‐mesh size, followed by extraction in 1200 L of distilled water at ambient temperature over 24 h. Similarly, 50 kg each of *L. chinense* and *E. ulmoides* were processed through grinding to 20 mesh and extracted together using 1200 L of distilled water at 60°C for 24 h. The extracts were then filtered through a 1 μm housing filter to remove particulates. The filtrates were mixed and concentrated under reduced pressure to a final concentration of 65 brix%. The resulting 110 kg concentrate was designated as Osteo‐F. A final product voucher specimen (Osteo‐F‐B2001‐1) was stored at BOINBIO Convergence Co. Ltd. and assigned the batch number B2001‐1. To ensure consistency and quality, Osteo‐F was standardized using high‐performance liquid chromatography (HPLC) for simultaneous quantification of three key marker compounds: 5‐hydroxymethyl‐2‐furaldehyde (5‐HMF), geniposidic acid, and schizandrin. Chromatographic analysis was carried out using a Thermo UHPLC Vanquish system and an Agilent 1200 series equipped with an XSelect analytical column (150 mm × 4.6 mm, 3.5 μm). The mobile phase was delivered at a flow rate of 1 mL/min, with detection performed at a wavelength of 254 nm and a column temperature of 25°C. The injection volume was set at 10 μL. The retention times for 5‐HMF, geniposidic acid, and schizandrin were 8.7, 12.8, and 24.5 min, respectively. Calibration curves for these markers exhibited strong linearity, with correlation coefficients (*R* ^2^) exceeding 0.9996. The standardized concentrations of 5‐HMF, geniposidic acid, and schizandrin in Osteo‐F were measured at 6.2, 2.0, and 0.5 mg/g, respectively, confirming the consistency and reproducibility of the formulation. These results validate the formulation as a reliable candidate for further applications (Lee et al. 2021).

### 2.2 Network Analysis of Osteo‐F

To elucidate the molecular interactions of Osteo‐F with potential targets in osteoarthritis (OA), a comprehensive network was constructed. Compounds constituting Osteo‐F were identified from relevant literature and detailed in Supporting Information. Chemical‐gene co‐occurrence data for these compounds were retrieved from the PubChem database. The STRING database was employed to aggregate the target proteins associated with these compounds, and the data were visualized and organized using the Cytoscape software version 3.9.2. Duplicate targets were removed to refine the network, and OA‐related genes were identified through searches in the DisGeNET database using ‘OA’ as a keyword. The analysis focused on identifying overlap between the genes associated with Osteo‐F and those linked to OA, with a particular emphasis on those with high relevance scores. Functional enrichment analysis was performed to link the Osteo‐F gene network with biological pathways and processes that could elucidate its therapeutic potential in OA. The KEGG pathway analysis was integrated using the Cytoscape environment to identify pathways significantly associated with the network. Gene Ontology (GO) analysis was also conducted to determine the involvement of enriched biological processes and signaling pathways. All statistical analyses were conducted to determine the significance of the enriched pathways and processes, with a threshold for significance set at *p*‐values less than 0.05.

### 2.3 Induction of MIA‐Induced Osteoarthritis

Five‐week‐old male Sprague–Dawley rats were acquired from RAON Bio (Yongin, Korea) and maintained under a 12‐h light/dark cycle at 20°C ± 5°C and 55% ± 15% humidity. Following a 2‐week acclimation period, the rats were allocated into six experimental groups: normal control (NOR), negative control (MIA), positive control (IDM), and three treatment groups (MIA + Osteo‐F 1 mg/kg [OF 1], MIA + Osteo‐F 10 mg/kg [OF 10], MIA + Osteo‐F 100 mg/kg [OF 100]), each comprising five rats. The test groups received an intra‐articular injection of monoiodoacetic acid (MIA, 3 mg/50 μL per rat), while the NOR group was administered an equivalent volume of saline. Following the initial MIA injection, treatments were administered for 4 weeks. Rats in the MIA groups were gavage‐fed daily with Osteo‐F at respective concentrations, whereas the MIA + Indo group received 2 mg/kg of indomethacin orally, and the NOR and MIA groups received distilled water orally. Each treatment was delivered in a volume of 200 μL per rat. Upon completion of the experiment, the femurs (left and right) were meticulously dissected to remove muscle tissue, and images of both the lateral and medial condyles were captured. Blind assessments were carried out by three experts, who scored the cartilage and bone health of both knees on a scale of 0–5 (*n* = 10). The scoring definitions are as follows: 0 indicates an intact articular surface; 1 indicates no more than 10 punctate depressions per condyle; 2 indicates more than 10 punctate depressions per condyle; 3 indicates erosion affecting up to 50% of the joint surface; 4 indicates erosion affecting more than 50% of the joint surface; and 5 indicates complete bone destruction (Udo et al. 2016). The protocol for these experiments was approved by the Committee on the Care and Use of Laboratory Animals at Kyung Hee University (KHSASP‐23‐027).

### 2.4 Bone Histology

The right femurs were first fixed in 10% neutral buffered formalin for 18 h to preserve tissue morphology, followed by demineralization in a 0.1 M ethylene diamine tetra‐acetic acid (EDTA) aqueous solution for 1 month to remove calcium deposits, facilitating subsequent sectioning. After complete demineralization, the femur samples were sequentially dehydrated using increasing concentrations of ethanol and then cleared with xylene to prepare them for embedding. The prepared samples were then embedded in paraffin wax, which provides a firm matrix for thin sectioning (10 μm). Sagittal sections of the femur were cut and mounted onto glass slides. These sections underwent a series of staining processes to highlight different histological features: hematoxylin and eosin (H&E) staining was employed to visualize the general tissue structure. Toluidine blue staining (#T3260, Sigma‐Aldrich) was used for its affinity to acidic tissue components. Lastly, Safranin‐O (#TMS‐009, Sigma‐Aldrich), a cationic dye, specifically stained the glycosaminoglycans in the cartilage red. Histological examination was performed using a Leica light microscope, part of the Leica Application Suite (LAS; Leica Microsystems, Buffalo Grove, IL, USA), allowing for detailed observation of tissue morphology. Digital images of the sections were captured at a magnification of ×100, enabling precise documentation and analysis of the histological changes observed.

### 2.5 Dual Energy X‐Ray Absorptiometry Test

Upon sacrifice, femurs were meticulously detached for analysis. The assessment of bone mineral content (BMC) and bone mineral density (BMD) for the left femurs was performed using dual‐energy X‐ray absorptiometry (DXA) with an InAlyzer system (Medikors, Seongnam, Korea).

### 2.6 Cell Culture

The experiment utilized the SW1353 human cartilage cell line, sourced from the American Type Culture Collection (ATCC, Manassas, VA, USA). This cell line was cultured in Dulbecco's Modified Eagle's Medium (DMEM, Welgene, Gyeongbuk, Korea), enriched with 10% fetal bovine serum (FBS, #S001‐01, Welgene, Gyeongbuk, Korea) and 1% penicillin–streptomycin (Gibco, Grand Island, NY, USA). The culturing environment was controlled at 37°C with a 5% CO_2_ atmosphere. Routine subculturing occurred 2–3 times per week, seeding 1.0 × 10^5^ cells into each 100 mm dish. In experiments involving IL‐1β, cells were exposed to IL‐1β (dissolved in PBS, #10010023, Gibco, USA), with a positive control of Loratadine 50 μM (Sigma‐Aldrich, USA) and doses of Osteo‐F at 1, 10, and 100 μg/mL. Cells were harvested 24 h post‐treatment, washed with ice‐cold PBS, centrifuged at 2000 *g* for 5 min at 4°C, and stored at −70°C for subsequent analyses.

### 2.7 Western Blotting Analysis

Protein extraction was performed using RIPA buffer (50 mM Tris–HCl, pH 7.4, 1% Nonidet P‐40, 0.5% sodium deoxycholate, 150 mM NaCl) supplemented with protease inhibitors (Roche, Hoffmann, USA). Proteins were quantified using the Bradford method. We prepared 20 μg of protein from each sample, denatured it in sodium dodecyl sulfate buffer, and transferred it to a PVDF membrane (Bio‐Rad, Hercules, CA, USA). The membrane was incubated with primary antibodies for NF‐κB (nucleus and cytosol), Lamin B, c‐Jun, p‐JNK, JNK, p‐IκB, IκB, and β‐actin, all diluted at 1:1000 in TBS‐T buffer. This was followed by a one‐hour incubation with secondary antibodies (Santa Cruz, CA, USA) at room temperature. Detection of protein bands was achieved using an enhanced chemiluminescence (ECL) kit (Amersham Pharmacia, Piscataway, NJ, USA).

### 2.8 RT‐PCR Analysis

Chondrocytes were isolated from the femoral cartilage for OA‐related marker analysis. The isolated chondrocytes were incubated with TRIzol reagent (Invitrogen, USA) at 4°C overnight, followed by thorough homogenization. Total RNA was then extracted according to the guidelines provided by Invitrogen, quantified, and reverse‐transcribed from 1 μg of RNA using Maxime RT Premix. The synthesized cDNA served as a template for PCR, utilizing Maxime PCR Premix (Invitrogen). Primers were designed based on Table 1. The levels of matrix metalloproteinase‐1 (MMP‐1), matrix metalloproteinase‐3 (MMP‐3), matrix metalloproteinase‐13 (MMP‐13), type II collagen (COL‐2), glyceraldehyde‐3‐phosphate dehydrogenase (GAPDH), tumor necrosis factor alpha (TNF alpha), interleukin 1 beta (IL1B), interleukin 4 (IL4), and interleukin 13 (IL13) were amplified. All bands were analyzed by Image J software.

**TABLE 1:** Primer sequences for RT‐PCR.

Species	Genes	Forward primers (5′–3′)	Reverse primers (5′–3′)
Mouse	*MMP1*	GCTGGGAGCAAACACATCTGAGGT	TGAGCCGCAACACGATGTAAGTTG
	*MMP3*	CTGTGTGTGGTTGTGTGCTCATCCTAC	GGCAAATCCGGTGTATAATTCACAATC
	*MMP13*	TGATGGACCTTCTGGTCTTCTGG	CATCCACATGGTTGGGAAGTTCT
	*COL2A1*	AGGGCCAGGATGTCCGGCA	GGGTCCCAGGTTCTCCATCT
	*TNFA*	TACTGAACTTCGGGGTGATTGGTCC	CAGCCTTGTCCCTTGAAGAGAACC
	*IL1B*	CAGGATGAGGACATGAGCACC	CTCTGCACACTCAAACTCCAC
	*IL4*	ATGGGTCTCAACCCCCAGC	GCTCTTTACGCTTTCCAGGAAGTC
	*IL13*	ACCACGGTCATTGCTCTCA	GTGTCTCGGACATGCAAGCT
	*GAPDH*	GGCATGGACTGTGGTCATGA	TTCACCACCATGGAGAAGGC
Human	*TNFA*	CAAGGGACAAGGCTGCCCCG	TAGACCTGCCCGGACTCC
	*IL4*	TGCCTCCAAGAACACAACTG	TCTTGGTTGGCTTCCTTCAC
	*IL13*	TGAGGAGCTGGTCAACATCA	CAGGTTGATGCTCCATACCAT
	*GAPDH*	CCATCACCATCTTCCAGGAG	CCTGCTTCACCACCTTCTTG

*Note:* This table lists the primer sequences employed for RT‐PCR analysis across mouse and human samples. For each gene, the species, gene name, forward primer sequence (5′–3′), and reverse primer sequence (5′–3′) are provided. The genes assessed include matrix metalloproteinases (*MMP1*, *MMP3*, *MMP13*), collagen (*COL2A1*), and various cytokines (*TNFA*, *IL1B*, *IL4*, and *IL13*) in both mouse and human samples, with GAPDH used as a reference gene in human samples.

### 2.9 Statistical Analysis

Statistical analyses were performed using PRISM 6.0 (GraphPad, Boston, MA, USA). Data are expressed as means ± SEM. To determine the statistical significance of differences among experimental groups, one‐way ANOVA was employed, followed by Tukey's multiple comparison tests as appropriate. In all cases, a *p*‐value of < 0.05 was established as the threshold for statistical significance.

## 3 Results

### 3.1 Osteo‐F Network and Shared Molecular Targets in Osteoarthritis

**FIGURE 1:**
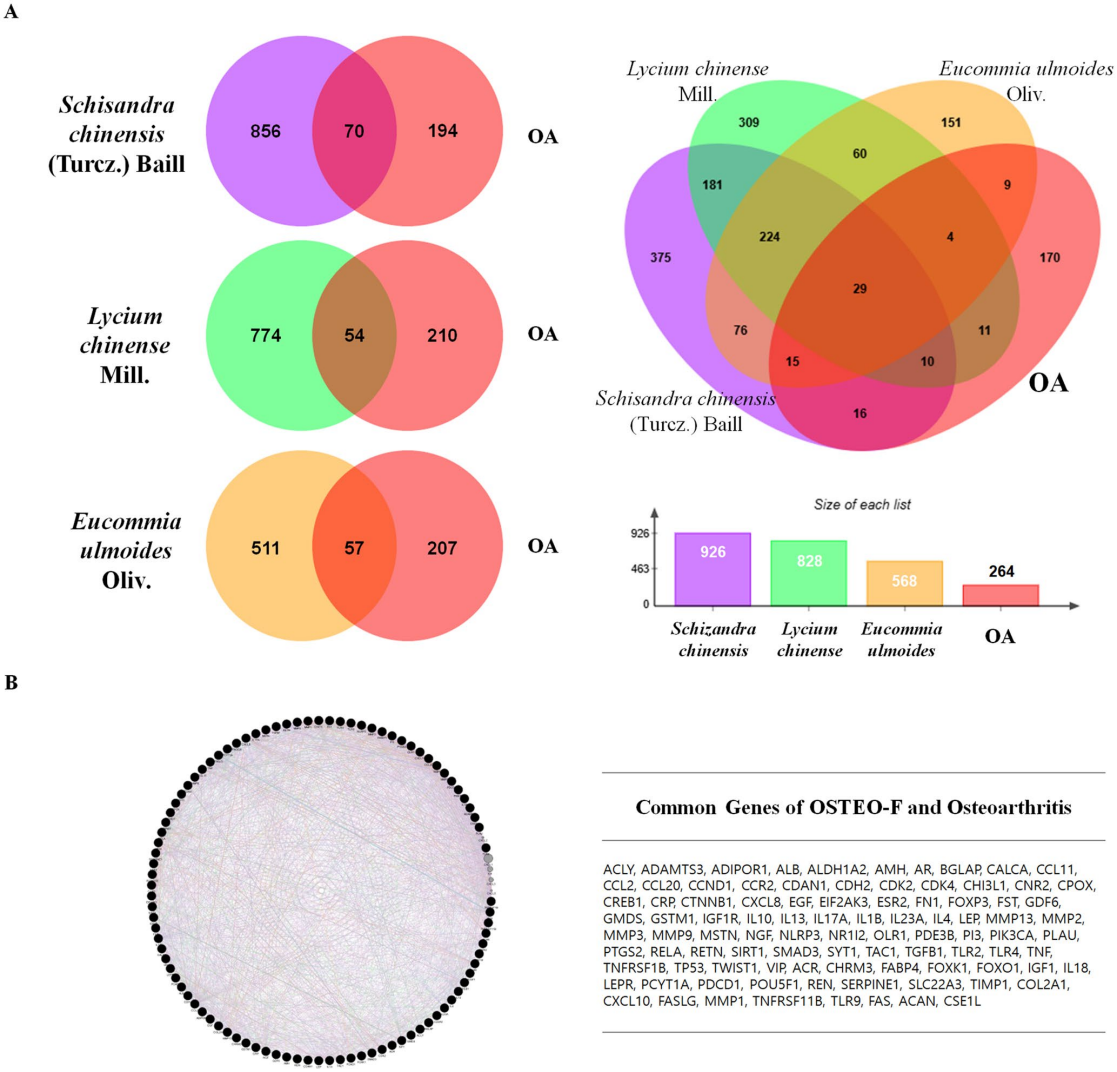
Identification of Osteo‐F network. (A) The Osteo‐F network was constructed by identifying the compounds and their co‐related genes within Osteo‐F ingredients. The network organized the main compounds, resulting in the identification of numerous targets after eliminating duplicates. Osteoarthritis‐related genes were gathered using the Disgenet database, and the overlapping genes between the *S. chinensis* , *L. chinense* , and *E. ulmoides* gene sets were prioritized. (B) The combined gene sets of *S. chinensis* , *L. chinense* , and *E. ulmoides* , with overlapping genes removed, revealed a unique set of target genes for Osteo‐F. Comparison with osteoarthritis‐associated genes showed a significant overlap, indicating that a substantial proportion of Osteo‐F target genes are associated with osteoarthritis.

The Osteo‐F network was constructed by utilizing compounds and co‐related genes of Osteo‐F ingredients. The Osteo‐F network was constructed by identifying compound‐related targets from 1877 main compounds, resulting in the identification of 8855 targets after eliminating duplicates. By searching for osteoarthritis (OA) as a keyword in the Disgenet database, we gathered OA‐related genes and counted the overlapping genes between the *S. chinensis* , *L. chinense* , and *E. ulmoides* gene set, prioritizing those with relevance scores exceeding 20. *S. chinensis* had 926 specific target genes, *L. chinense* had 828, and *E. ulmoides* had 568 (Figure 1A). When combined and overlapping genes were removed, Osteo‐F had 8855 unique target genes, while OA was associated with 264 genes. When comparing these two sets of genes, 217 of them were found to be the same in both. This indicates that approximately 36.36% of the target genes for Osteo‐F overlap with the target genes associated with OA (Figure 1B).

### 3.2 KEGG and GO Enrichment Analysis of Osteo‐F Compounds

Incorporating KEGG pathway enrichment analysis into the functional enrichment analysis of the Osteo‐F gene network using Cytoscape, we discovered a correlation between the Osteo‐F network and the KEGG OA‐related pathway. This association led to the identification of matched genes that potentially contribute to the therapeutic potential of Osteo‐F. Moreover, our analysis highlighted the involvement of the ‘Osteoclast differentiation’, ‘TNF signaling pathway’, ‘IL‐17 signaling pathway’, ‘PI3K‐Akt signaling pathway’, ‘NF‐kappa B signaling pathway’, and ‘JAK‐STAT signaling pathway’ in the mechanism of Osteo‐F in OA. Additionally, the GO process analysis revealed significant enrichment in processes such as ‘positive regulation of response to stimulus’, ‘response to cytokine’, ‘inflammatory response’, ‘cytokine‐mediated signaling pathway’, ‘response to growth factor’, ‘response to wounding’, and ‘tissue remodeling’ (*p*‐value < 0.05) (Table 2).

**TABLE 2:** Osteo‐F target pathway based on KEGG 2021 human pathway and GO biological process.

Category	Description	*p* (< 0.05)	Background genes	Common genes
KEGG pathway	TNF signaling pathway	3.97E‐16	111	19
	Cytokine‐mediated signaling pathway	6.09E‐14	120	16
	Response to wounding	6.22E‐13	158	14
	Osteoclast differentiation	2.83E‐12	101	11
GO biological process	Inflammatory response	6.32E‐37	538	57
	Positive regulation of response to stimulus	9.34E‐36	2131	98
	Response to growth factor	4.34E‐28	503	47
	Response to cytokine	1.97E‐22	804	50
	Cytokine‐mediated signaling pathway	3.76E‐17	369	27
	Response to wounding	2.59E‐16	444	28
	Osteoclast differentiation	3.97E‐16	101	14

*Note:* The table displays the enriched KEGG pathways and GO biological processes associated with Osteo‐F target genes based on KEGG 2021 human pathway and GO biological process analyses. The categories include various signaling pathways and biological processes relevant to osteoarthritis, indicating the potential mechanisms through which Osteo‐F exerts its therapeutic effects. The *p*‐values represent the significance of the enrichment, with a threshold of *p* < 0.05. The background genes column indicates the total number of genes in each pathway or process, while the common genes column lists the number of genes from the Osteo‐F network that are involved in each pathway or process.

### 3.3 Effects of Osteo‐F on the Destruction of Cartilage and Arthritis Scores in Intra‐Articular Injection of MIA‐Induced Rats

**FIGURE 2:**
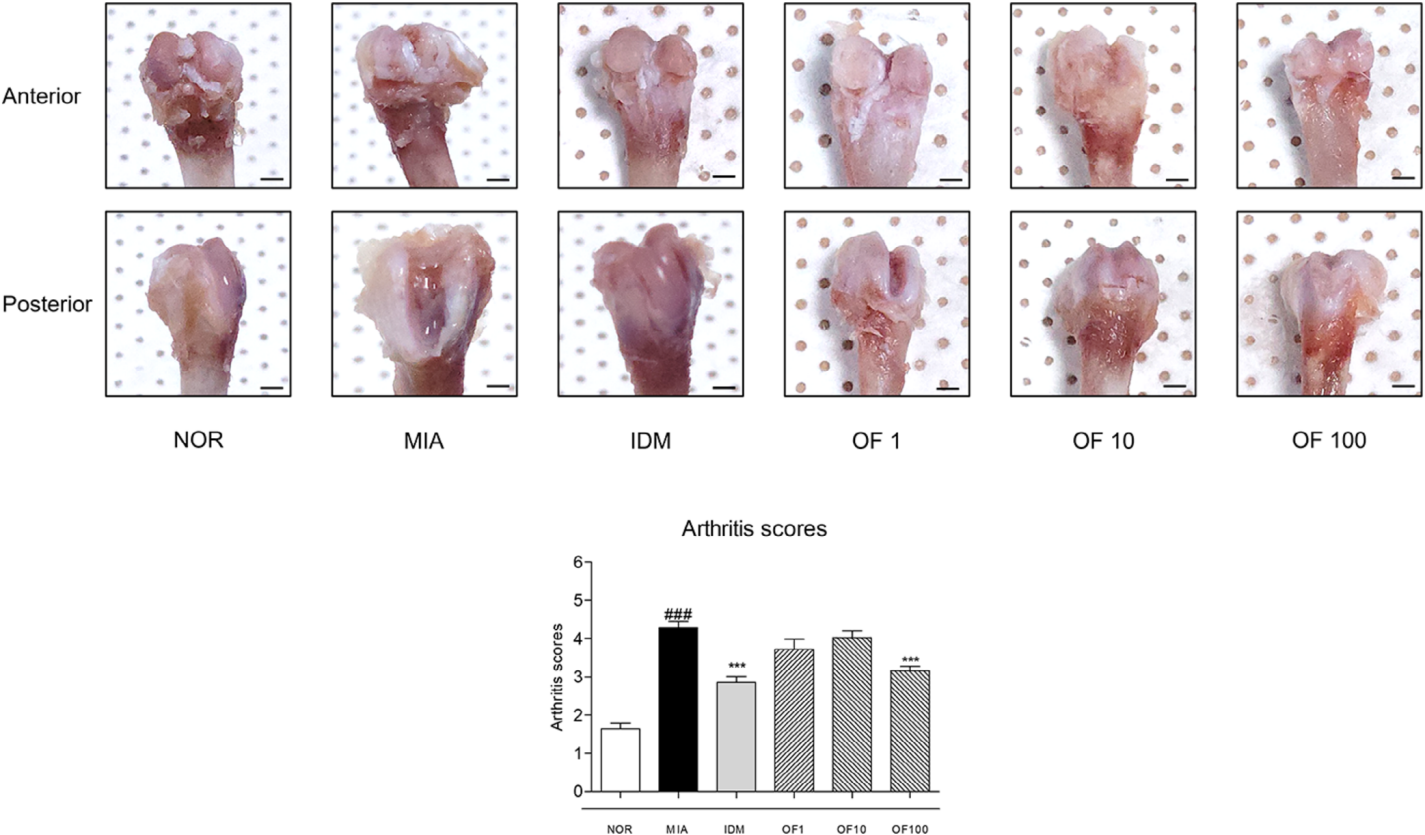
Effects of Osteo‐F on cartilage destruction and arthritis scores in MIA‐induced rats. Representative images of the anterior and posterior sides of the epicondyle in the knee joint. Data are expressed as the means ± SD. ^###^ *p* < 0.001 compared to the NOR group; ***p < 0.001 compared to the MIA group. IDM, indomethacin; MIA, monosodium iodoacetate; OF 1, Osteo‐F 1 mg/kg; OF 10, Osteo‐F 10 mg/kg; OF100, Osteo‐F 100 mg/kg.

In the OA NOR group, rats exhibited an arthritis score of 4.29 ± 0.60, indicative of moderate OA pathology characterized by significant proteoglycan depletion and cartilage erosion. Conversely, rats treated with 100 mg/kg Osteo‐F showed significantly improved conditions, achieving a lower arthritis score of 3.16 ± 0.33 (OF 100), suggesting reduced proteoglycan loss and lesser cartilage damage. Macroscopic evaluations of tibial and femoral cartilage further confirmed these observations; pronounced cartilage defects were evident in the untreated OA group, whereas they were notably diminished in the Osteo‐F‐treated group. These findings suggest that Osteo‐F treatment effectively slows OA progression, as evidenced by comprehensive cartilage integrity evaluations both microscopically and macroscopically (Figure 2).

### 3.4 Effects of Osteo‐F on the Structure of Cartilage in Intra‐Articular Injection of MIA‐Induced Osteoarthritis Rats

**FIGURE 3:**
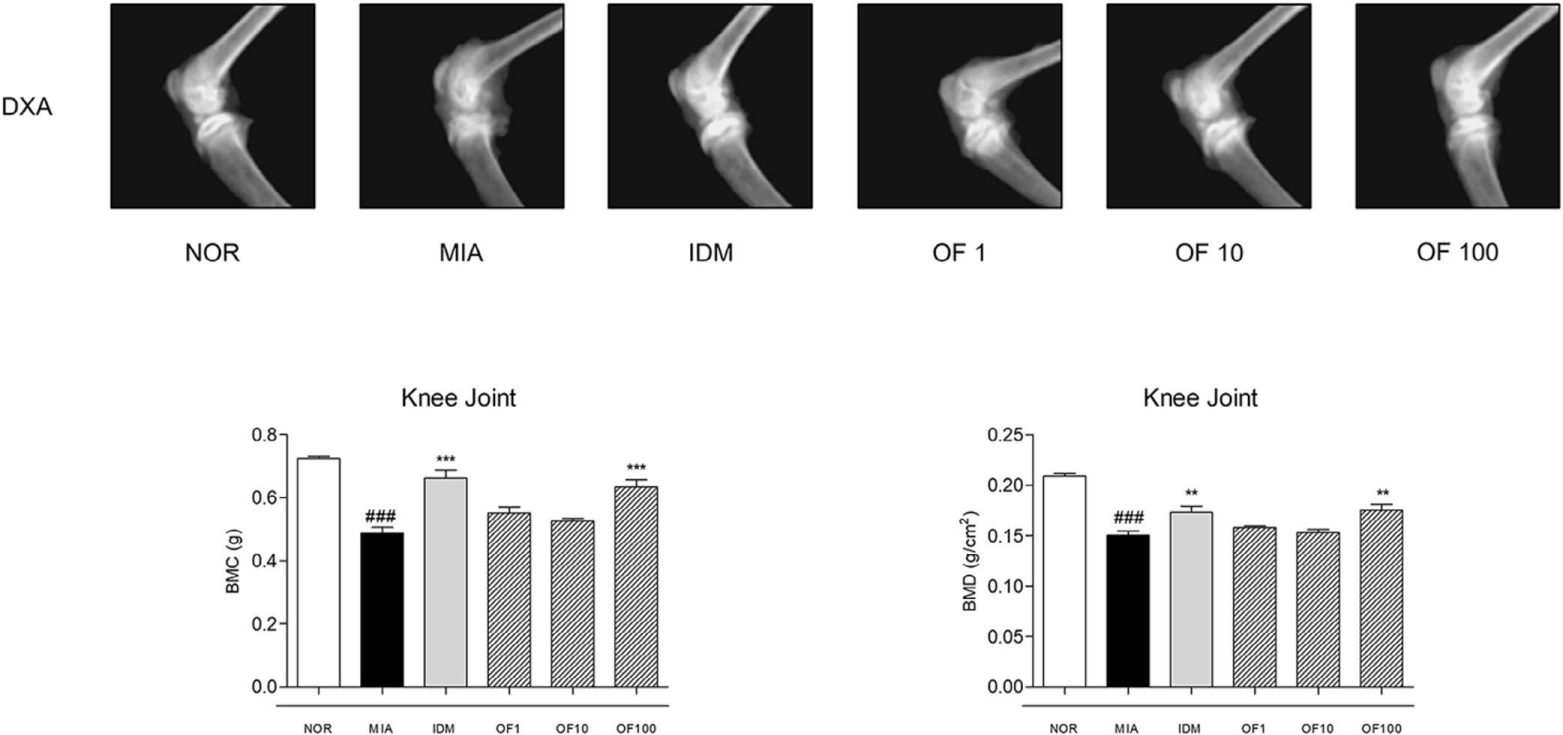
Effects of Osteo‐F on levels of BMC and BMD in MIA‐induced rats. Images present the levels of BMC and BMD in the knee joint, as measured by DXA. Data are expressed as the means ± SD. ^###^ *p* < 0.001 compared to the NOR group; **p < 0.01 and ***p < 0.001 compared to the MIA group. BMC, bone mineral content; BMD, bone mineral density; DXA, dual energy X‐ray absorptiometry; IDM, indomethacin; MIA, monosodium iodoacetate; OF 1, Osteo‐F 1 mg/kg; OF 10, Osteo‐F 10 mg/kg; OF100, Osteo‐F 100 mg/kg.

BMC and BMD in the femurs of MIA‐induced osteoarthritic rats were meticulously quantified using DXA, with results compared to those of a reference group. Osteoarthritic rats exhibited a significantly lower femoral BMC of 0.4868 ± 0.0471 g/cm^2^, compared to 0.7243 ± 0.0172 g/cm^2^ in the reference group. Treatment with 100 mg/kg Osteo‐F resulted in a 30.16% increase in femoral BMC from the osteoarthritic baseline. IDM treatment improved femoral BMC to 0.6624 ± 0.0664 g/cm^2^ and showed a 36.07% increase compared to the MIA group. Femoral BMD was reduced by 38.99% in osteoarthritic rats, compared to 0.2089 ± 0.0102 g/cm^2^ in the MIA group. Treatment with varying doses of Osteo‐F significantly elevated femoral BMD, with the highest dose (OF100) showing the most pronounced improvements. IDM treatment further raised femoral BMD to 0.1728 ± 0.0225 g/cm^2^. Collectively, 100 mg/kg Osteo‐F treatment facilitated significant recoveries of 41.98% in BMC and 61.81% in BMD, confirming its therapeutic efficacy in this model (Figure 3).

### 3.5 Effects of Osteo‐F on the Histological Changes in Intra‐Articular Injection of MIA‐Induced Rats

**FIGURE 4:**
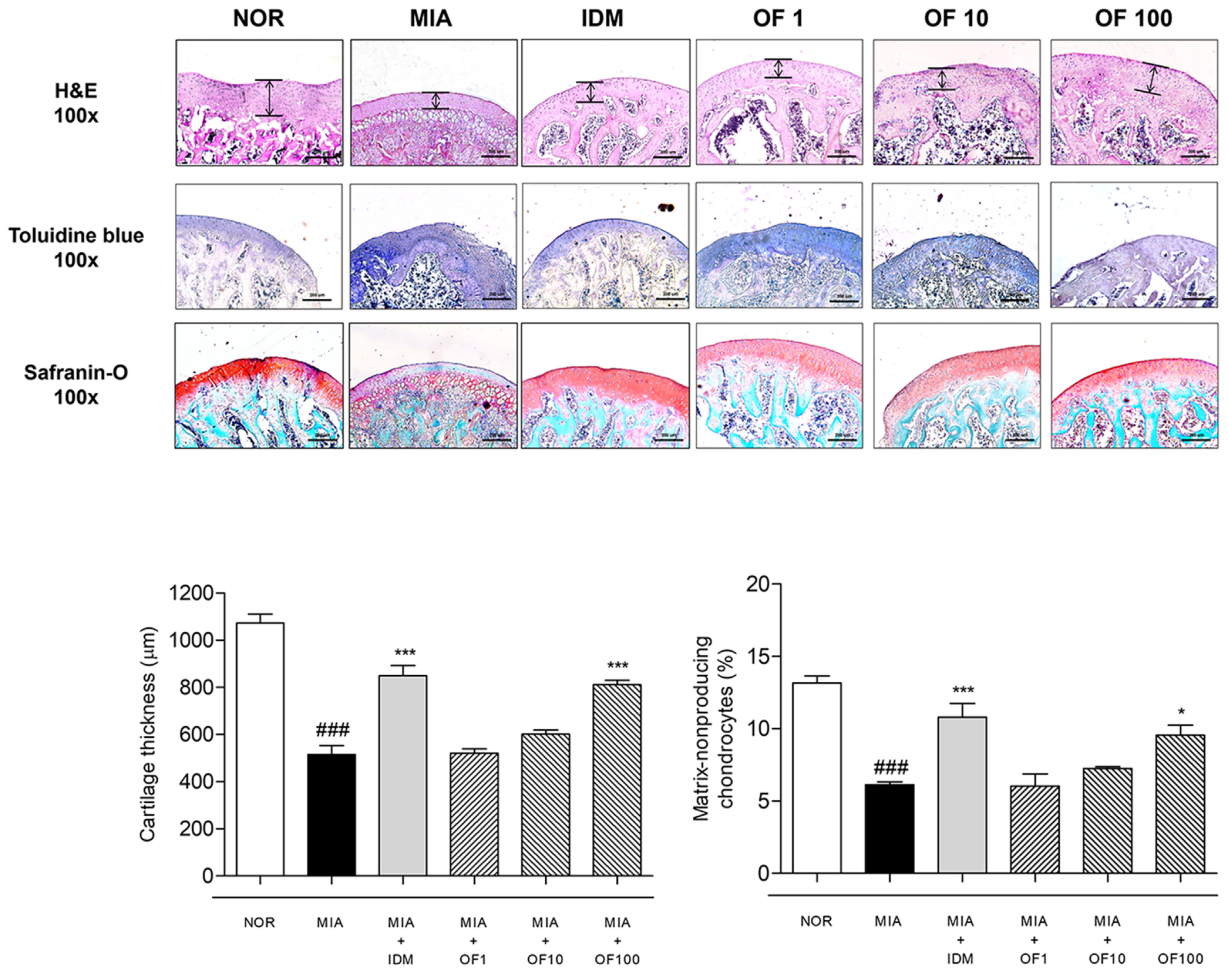
Histological analysis of femoral articular cartilage in MIA‐induced rats. This figure displays images of femoral articular cartilage from MIA‐induced osteoarthritis rats, stained with H&E, safranin O, and toluidine blue. H&E staining reveals the chondrocyte population within the cartilage, safranin O staining accentuates the proteoglycan content, and toluidine blue staining offers insights into the distribution and levels of proteoglycans. Each image is captured with a scale bar representing 200 μm and a magnification of ×100. H&E, hematoxylin and eosin; IDM, indomethacin; MIA, monosodium iodoacetate; OF 1, Osteo‐F 1 mg/kg; OF 10, Osteo‐F 10 mg/kg; OF100, Osteo‐F 100 mg/kg. ### *p* < 0.001 compared to the NOR group; *p < 0.05 and ***p < 0.001 compared to the MIA group.

Histological analysis of femoral articular cartilage from MIA‐induced osteoarthritic rats revealed substantial alterations compared to the NOR group. In H&E‐stained sections, the articular cartilage thickness of the NOR group was 1072.0 ± 38.15 μm, whereas the MIA group exhibited a severe reduction to 515.8 ± 37.83 μm (^###^ *p* < 0.001 vs. NOR). Osteo‐F treatment at 1, 10, and 100 mg/kg resulted in partial recovery to 849.1 ± 43.67, 520.0 ± 19.36, 601.5 ± 18.34, and 811.2 ± 18.86 μm, respectively (*p* < 0.001 vs. MIA for OF 100). In contrast, treatment with Osteo‐F, particularly at 100 mg/kg, significantly improved proteoglycan retention as demonstrated by Toluidine Blue staining. The staining intensity score increased from 6.120 ± 0.20 in the MIA group to 9.563 ± 0.68 in the Osteo‐F 100 mg/kg group (*p < 0.05 vs. MIA), while the NOR group showed a baseline of 13.16 ± 0.48 (^###^ *p* < 0.001 vs. MIA), indicating substantial restoration of the cartilage matrix. Safranin O staining showed a similar visual trend, in which the MIA group displayed marked depletion of proteoglycans, particularly in the deeper layers of cartilage, whereas Osteo‐F at 100 mg/kg preserved overall matrix integrity and staining intensity. Although no quantification was performed for Safranin O staining, the visual improvement supported the findings observed with Toluidine Blue (Figure 4).

### 3.6 Effects of Osteo‐F on the Expression of Osteoarthritis‐Related mRNA in Cartilage of MIA‐Induced Rats

**FIGURE 5:**
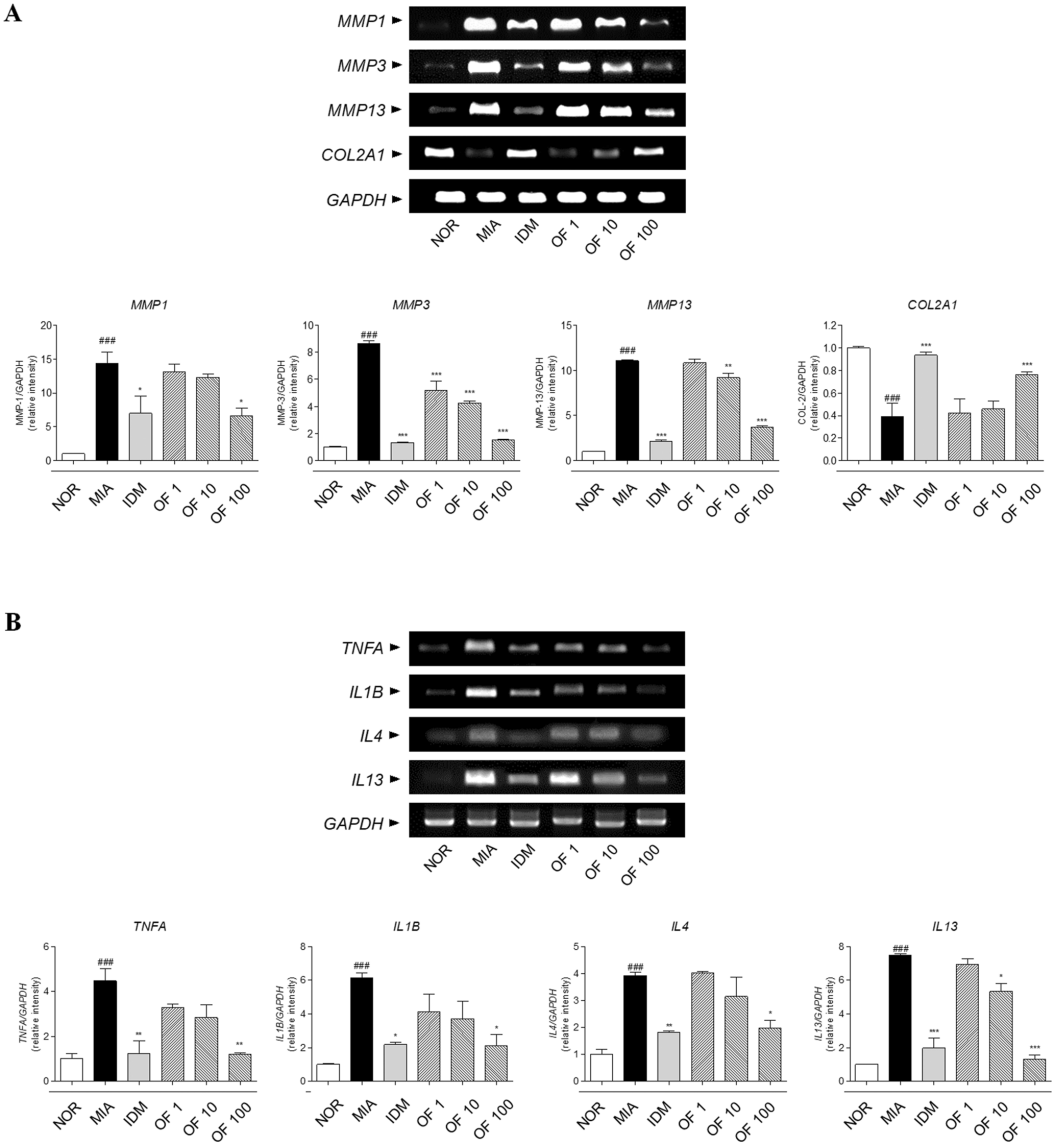
mRNA expression levels of OA‐related markers in MIA‐induced rats. (A) This figure displays the mRNA expression levels of *MMP1*, *MMP3*, *MMP13*, and *COLIIA1* in chondrocyte tissues, quantified using RT‐PCR. (B) The figure shows the expression levels of *IL4*, *IL13*, *TNFA*, and *IL1B* in chondrocyte tissues. Data are expressed as the means ± SD. ^###^ *p* < 0.001 compared to the NOR group; *p < 0.05, **p < 0.01, and ***p < 0.001 compared to the MIA group. COL2A1, type II collagen A1; IDM, indomethacin; IL, interleukin; MIA, monosodium iodoacetate; MMP, matrix metalloproteinase; OF 1, Osteo‐F 1 mg/kg; OF 10, Osteo‐F 10 mg/kg; OF100, Osteo‐F 100 mg/kg; RT‐PCR, reverse transcription polymerase chain reaction.

In the MIA‐induced OA model, mRNA expression levels of MMP‐1, MMP‐3, and MMP‐13 were significantly elevated, showing increases of 14.35‐, 8.63‐, and 11.06‐fold, respectively, compared to the NOR group. Treatment with indomethacin significantly reduced the expression levels of these enzymes in chondrocyte tissues by 51.36%, 84.68%, and 80.74%, respectively, relative to the MIA group. Similarly, treatment with 100 mg/kg Osteo‐F significantly decreased the expression of MMP‐1, MMP‐3, and MMP‐13 by 54.26%, 82.56%, and 66.60%, respectively (*p* < 0.001). Additionally, COL‐2 expression was reduced to 0.3875‐fold in the MIA group compared to the NOR group. This reduction was counteracted by indomethacin treatment, which increased COL‐2 expression by 141.23% relative to the MIA condition. The 100 mg/kg dosage of Osteo‐F also significantly upregulated COL‐2 expression by 97.1% in chondrocyte tissues (*p* < 0.001) (Figure 5A). Furthermore, significant differences (*p* < 0.01) were observed in the mRNA expressions of inflammatory cytokines between the NOR and MIA‐induced rat chondrocytes. IL‐4, IL‐13, TNF‐α, and IL‐1β levels were elevated approximately 14.80‐, 5.48‐, 4.6‐, and 6.16‐fold, respectively, following MIA induction. These levels were notably reduced by 67.57%, 62.83%, 62.8%, and 65.5%, respectively, after treatment with 100 mg/kg Osteo‐F (*p* < 0.005) (Figure 5B).

### 3.7 Effects of Osteo‐F on the Expression of Inflammation Factors in the IL‐1β‐Induced SW1353 Cell Line

**FIGURE 6:**
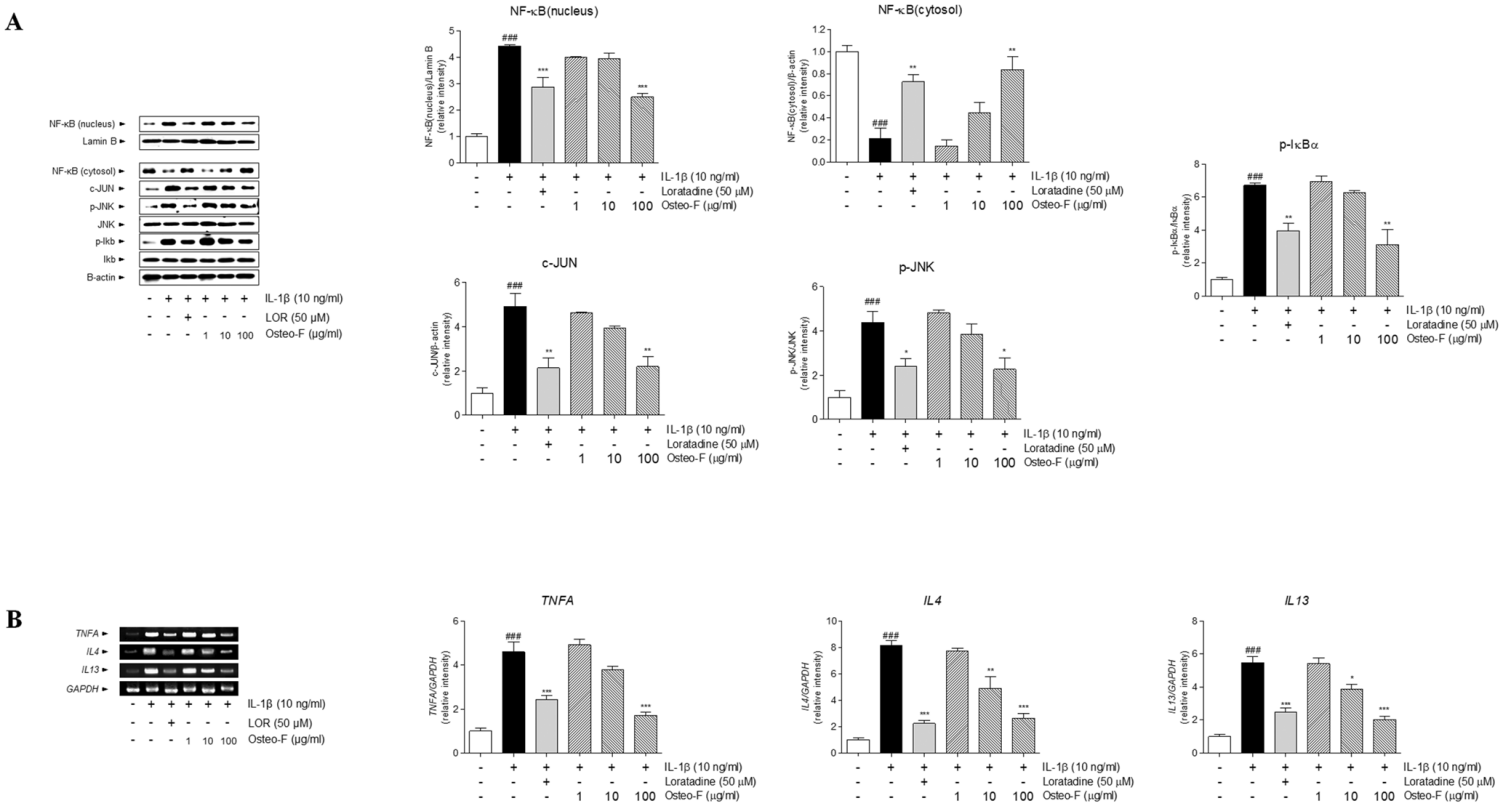
Modulation of inflammatory pathways in SW1353 chondrocytes by Osteo‐F. (A) The effects of Osteo‐F on the JNK/NF‐κB/IκB‐α signaling pathway activation in SW1353 chondrocytes treated with IL‐1β. (B) The figure includes RT‐PCR analysis of the expression levels of pro‐inflammatory cytokines *TNFA*, *IL4*, and *IL13* in SW1353 chondrocytes treated with IL‐1β. Data are expressed as the means ± SD. ^###^ *p* < 0.001 compared to non‐treated cells; *p < 0.05, **p < 0.01, and ***p < 0.001 compared to IL‐1β treated cells. c‐Jun, c‐transcription factor Jun; IDM, indomethacin; IκB, inhibitor of kappa B; JNK, c‐Jun N‐terminal kinase; MIA, monosodium iodoacetate; NF‐κB, nuclear factor kappa‐light‐chain‐enhancer of activated B cells; OF 1, Osteo‐F 1 mg/kg; OF 10, Osteo‐F 10 mg/kg; OF100, Osteo‐F 100 mg/kg.

Significant changes were observed in the phosphorylation of c‐Jun N‐terminal kinase (JNK) in IL‐1β‐treated SW1353 cells compared to untreated controls. Specifically, JNK phosphorylation was significantly enhanced in cells solely exposed to IL‐1β, but this effect was notably reduced when cells were treated with Osteo‐F at concentrations of 1, 10, and 100 μg/mL. Additionally, IL‐1β‐induced upregulation of the transcription factor c‐JUN was substantially decreased in cells treated with 100 μg/mL of Osteo‐F. This treatment also significantly lowered the levels of phosphorylated JNK (p‐JNK), demonstrating Osteo‐F's regulatory influence on this signaling pathway. Furthermore, IL‐1β treatment resulted in elevated levels of nuclear NF‐κB and increased cytosolic phosphorylation of IκB‐α, with rises of 4.44 and 6.71 times, respectively. Conversely, IL‐1β treatment unexpectedly reduced cytosolic NF‐κB levels by approximately 78%. Treatment with 100 μg/mL of Osteo‐F countered these effects by decreasing nuclear NF‐κB levels by 43.5% and increasing cytoplasmic NF‐κB by 297.67%. Additionally, this treatment led to a 53.6% reduction in phosphorylated IκB‐α levels in the cytosol compared to IL‐1β‐only treated cells (Figure 6A). Exposure to IL‐1β also induced a significant increase in mRNA levels of inflammatory cytokines in SW1353 cells. Specifically, TNF‐α, IL‐4, and IL‐13 expression levels rose by 4.59, 8.13, and 5.48‐fold, respectively, in comparison to untreated cells. Remarkably, treatment with 100 μg/mL of Osteo‐F significantly reduced these elevated levels of TNF‐α by 62.8%. Moreover, after 24 h of exposure to IL‐1β, mRNA expressions of IL‐4 and IL‐13 were significantly decreased by 67.57% and 62.83%, respectively, following treatment with 100 μg/mL of Osteo‐F (Figure 6B).

## 4 Discussion

Osteoarthritis (OA) is a degenerative joint condition characterized by cartilage degradation, inflammation, and subchondral bone remodeling. These changes not only impair joint function but also significantly impact quality of life. Interestingly, OA and osteoporosis share overlapping mechanisms, including progressive subchondral bone loss and systemic inflammation, suggesting that therapeutic strategies targeting bone health could be effective in managing OA (Qu et al. 2023). Furthermore, dietary interventions and bioactive compounds have emerged as promising approaches for mitigating OA symptoms due to their multi‐targeted effects and safety profiles compared to conventional pharmacological treatments.

In this study, we evaluated a novel herbal‐based formulation, developed using food‐derived bioactive components, for its potential to improve joint health and mitigate OA symptoms. Previous research demonstrated the formulation's efficacy in improving bone health in osteoporosis models by upregulating osteogenic markers such as BMP‐2 and OPN (Lee et al. 2017; Wang et al. 2023). Building on these findings, the current study focused on its application in OA, emphasizing its ability to reduce cartilage degradation, inflammation, and bone remodeling.

A multi‐faceted methodological approach, including in silico network pharmacology, in vivo animal models, and in vitro cellular experiments, was employed to evaluate the formulation's therapeutic potential. Network pharmacology analysis revealed significant molecular targets shared between the formulation and OA, highlighting its potential to regulate key inflammatory and cartilage‐related pathways. Enrichment analysis identified associations with osteoclast differentiation and inflammatory signaling pathways, suggesting that the formulation could address both cartilage and bone deterioration in OA.

Our study was based on network pharmacology analysis to unravel the complex interactions characteristic of OA, a disease influenced by a multitude of genes and pathways (Wang et al. 2023). This approach is essential for diseases like OA, which are well‐known for the limitations of single‐target drugs, and supports our goal of describing the comprehensive mechanisms and therapeutic targets of the herb (Xiang et al. 2022; Zhang et al. 2022). The Osteo‐F network, illustrating shared molecular targets with OA, demonstrates the potential of Osteo‐F to influence key pathways involved in the disease pathogenesis. Discovering 217 overlapping genes between Osteo‐F targets and OA‐related genes offers promising insights into the molecular mechanisms driving its therapeutic effects. Enrichment analyses further corroborate that the Osteo‐F network is associated with essential biological pathways implicated in OA, such as osteoclast differentiation and several inflammatory signaling pathways. These findings articulate a mechanistic justification for the observed improvements in bone and cartilage health and highlight Osteo‐F's potential to mitigate the multifaceted characteristics of OA.

Building on the predictions from in silico network pharmacology analysis, we conducted pre‐clinical experiments using a MIA‐induced model to confirm Osteo‐F efficacy in mitigating degenerative processes of OA (Pitcher et al. 2016). Clinically, OA presents a spectrum of symptoms quantified by the arthritis score, a critical measure reflecting the severity of cartilage damage, joint pain, and stiffness (Kraus et al. 2015). In our study, observed reductions in arthritis scores among Osteo‐F treated groups, compared to the MIA group, provide robust evidence of its therapeutic advantages. Furthermore, it was confirmed that BMC and BMD were significantly improved after Osteo‐F treatment. These improvements are critical as BMC and BMD serve as essential markers of bone health and strength, which are often diminished in OA patients (Chen et al. 2022; Pouresmaeili et al. 2018). Therefore, these improvements indicate that Osteo‐F can directly address the key physical symptoms of OA by effectively reducing joint deterioration and improving joint function.

Additionally, to assess joint tissue morphology and structural integrity, we utilized H&E staining. This method is essential for visualizing cartilage general architecture and cellular organization (Decker et al. 2015). Treatment with Osteo‐F showed reduced erosion of the cartilage surface and maintained cellular organization, underscoring its role in preserving the structural integrity of cartilage compromised by OA. Proteoglycan, an essential component of the cartilage extracellular matrix, was highlighted using Safranin‐O staining. The depletion of these proteoglycans is a well‐known pathological feature of OA, leading to weakened cartilage and impaired joint function (Alcaide‐Ruggiero et al. 2023). Our findings demonstrate that Osteo‐F treatment significantly preserved proteoglycan content compared to the MIA group, indicating its protective effect against the degradation of critical cartilage components. Furthermore, we used Toluidine Blue staining to measure the glycosaminoglycan content in the cartilage. Glycosaminoglycan is an important component for maintaining the structure and function of cartilage (Jin et al. 2023). Our study showed that Osteo‐F helped maintain glycosaminoglycan levels, further affirming its role in preserving cartilage health and function in the context of OA.

Following the noted downregulation of cartilage matrix elements in our histological analyses, we expanded our research to molecular assessments, specifically measuring MMP expression in chondrocytes treated with Osteo‐F. The breakdown of cartilage, a hallmark of OA pathogenesis, is predominantly mediated by MMPs. These enzymes degrade essential components of the cartilage extracellular matrix, such as Type II collagen (Man and Mologhianu 2014). Type II collagen, a major structural protein in cartilage, endows it with tensile strength and integrity, while proteoglycans are crucial for its resilience and compressive strength (Sophia Fox et al. 2009). Osteo‐F enhancement of Type II collagen production may contribute to the maintenance or even regeneration of the cartilage matrix. The reduced expression of MMP‐1, MMP‐3, and MMP‐13 in chondrocytes suggests that Osteo‐F could protect against cartilage degradation. By inhibiting these MMPs, Osteo‐F appears to support cartilage integrity and thwart further deterioration, tackling the fundamental breakdown processes characteristic of OA, critical for preserving joint function and reducing pain. Moreover, its potential to preserve or restore proteoglycans could help maintain the biomechanical properties essential for healthy cartilage, which is vital for ensuring the structural and functional preservation of joints afflicted by OA.

**FIGURE 7:**
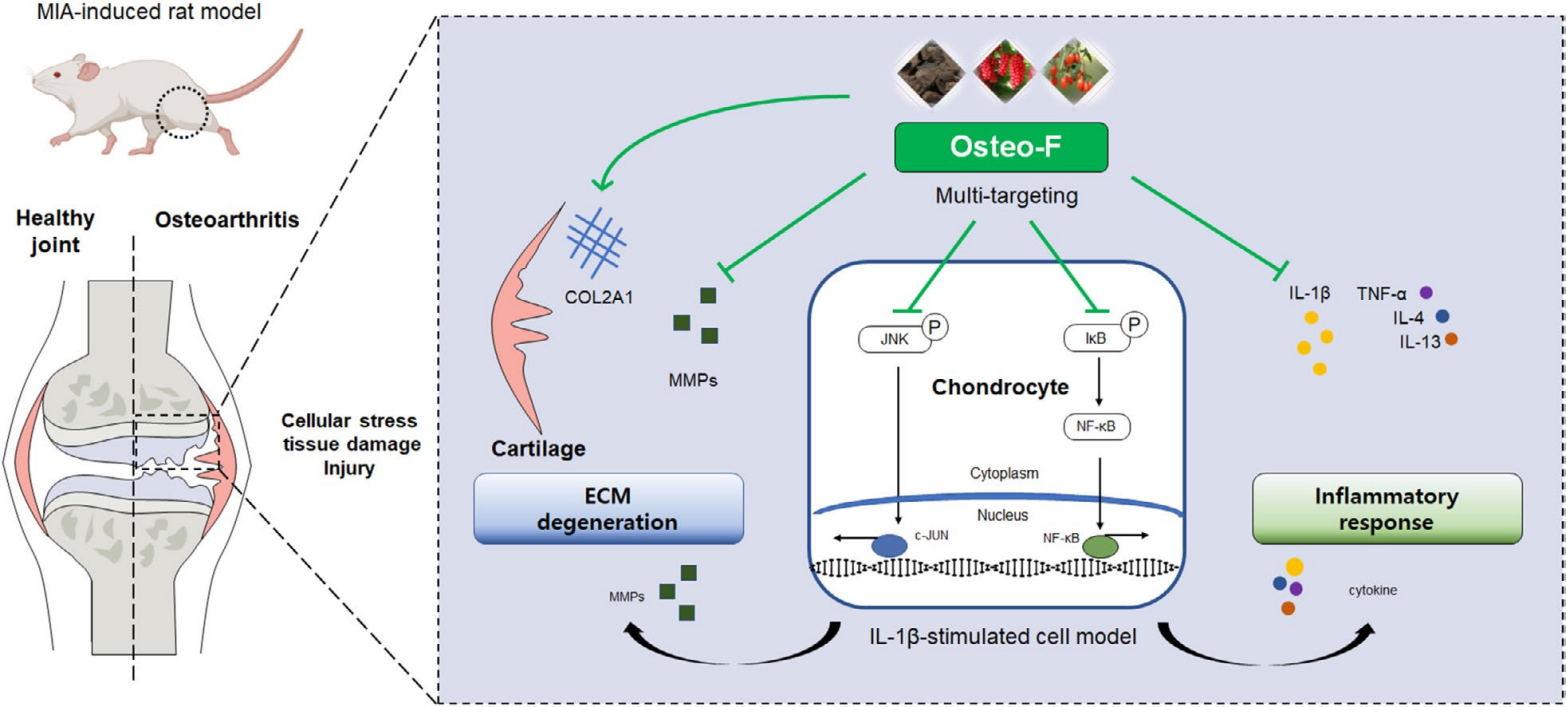
Mechanism of action of Osteo‐F in osteoarthritis. Figure illustrates the multifaceted mechanism by which Osteo‐F mitigates OA. It encapsulates how Osteo‐F interacts with various molecular and cellular pathways to address the complex pathology of OA. COL, collagen; IL, interleukin; MMP, matrix metalloproteinase.

Furthermore, OA is notably marked by chronic inflammation, mediated by high levels of pro‐inflammatory cytokines including IL‐1β, TNF‐α, IL‐4, and IL‐13, which significantly contribute to the disease progression (Sokolove and Lepus 2013). Our research demonstrated that Osteo‐F significantly reduced the levels of these cytokines within chondrocytes, indicating a strong anti‐inflammatory action. This finding is pivotal as these cytokines play key roles in OA pathogenesis, driving pain, swelling, and joint destruction. To delve deeper into the underlying mechanisms, we conducted experiments using SW1353 cells, a human chondrosarcoma cell line frequently employed to model chondrocyte behavior in OA. By inducing an inflammatory response in these cells with IL‐1β, which mimics the OA inflammatory milieu by elevating various inflammatory and catabolic markers (Lu et al. 2023), we compared the effects of Osteo‐F with loratadine—a drug commonly used in OA treatment (Hunto et al. 2020). Upon Osteo‐F treatment, we observed a downregulation of crucial proteins in the inflammatory pathway, including c‐Jun, phosphorylated JNK (p‐JNK), NF‐κB, and phosphorylated inhibitor of kappa B (p‐IκB). These changes suggest that Osteo‐F mitigates inflammation by inhibiting the JNK pathway and preventing NF‐κB from entering the nucleus, thereby decreasing the transcription of pro‐inflammatory genes. We validated the anti‐inflammatory properties of Osteo‐F at the mRNA level by documenting reduced expressions of TNF‐α, IL‐4, and IL‐13 in Osteo‐F‐treated cells. This downregulation confirms the anti‐inflammatory and anti‐catabolic actions of Osteo‐F, demonstrating its effectiveness in reducing inflammation and preventing cartilage degradation in OA. These results are significant, providing a molecular underpinning for the anti‐inflammatory and cartilage‐protective impacts of Osteo‐F seen in our earlier histological studies. By influencing key signaling molecules and cytokines central to OA pathogenesis (Yao et al. 2023), Osteo‐F promises a multifaceted strategy to alleviate inflammation and cartilage breakdown, addressing both the pain and structural challenges of the disease. Our thorough examination of Osteo‐F actions at the cellular and molecular levels demonstrates its potential as a promising therapeutic agent for OA (Figure 7).

Despite these promising results, some discrepancies were observed between the findings in in vivo and in vitro models. These differences may stem from intrinsic differences in cell type, as SW1353 cells are derived from human chondrosarcoma and differ from primary chondrocytes in their response to inflammatory stimuli. Additionally, the experimental conditions, including the use of IL‐1β stimulation for SW1353 cells and MIA induction in the animal model as well as differences in treatment durations, likely contributed to the variability. Previous research has shown that SW1353 cells may not fully replicate the behavior of primary chondrocytes, particularly in their response to cytokines such as IL‐1β, emphasizing the importance of validating findings across diverse models (Gebauer et al. 2005).

Furthermore, chemical profiling of the formulation identified schizandrin, betaine, and geniposidic acid as its primary bioactive components. Schizandrin, a dibenzocyclooctadiene lignan, has been reported to exhibit anti‐inflammatory and antioxidant properties, potentially contributing to cartilage protection (Tu et al. 2019). Betaine, known for its role in osmoregulation and cellular homeostasis, has demonstrated anti‐inflammatory effects that may support joint health (Zhao et al. 2018). Geniposidic acid, a bioactive iridoid glycoside, has been associated with anti‐inflammatory and cartilage‐protective activities in preclinical models (Sun et al. 2024). While these compounds likely play a significant role in the formulation's efficacy, the possibility of synergistic effects among the components warrants further investigation. High‐throughput analytical techniques, such as metabolomics, could provide deeper insights into the interactions between these components and their combined effects on joint health.

## 5 Conclusions

The impact of Osteo‐F on OA is multifaceted, addressing not only the symptoms but also the underlying mechanisms of disease progression. Osteo‐F may contribute to the treatment of joint disorders with its unique composition and diverse targeted approach. Continued exploration of Osteo‐F's effects on specific biomarkers and pathways will provide deeper insights into the molecular intricacies of OA and how they can be effectively targeted for more comprehensive management strategies.

## Author Contributions

**Seong Chul Jin:** data curation (lead), formal analysis (lead), investigation (lead), methodology (lead), resources (lead), software (lead), validation (lead), visualization (lead), writing – original draft (lead), writing – review and editing (equal). **You Yeon Choi:** formal analysis (equal), investigation (equal), validation (equal), writing – review and editing (lead). **Minwoo Song:** formal analysis (equal), investigation (equal). **Hee Kyung Baek:** formal analysis (equal), investigation (equal). **Seungyob Yi:** formal analysis (equal). **Eun‐Jung Kim:** funding acquisition (lead), supervision (equal). **Woong Mo Yang:** conceptualization (lead), methodology (lead), project administration (lead), supervision (lead).

## Conflicts of Interest

The authors declare no conflicts of interest.

## Supporting information


Appendix S1

Functional enrichment analysis (Common genes of Osteo‐F and Osteoarthritis)

## Data Availability

The data that support the findings of this study are available on request from the corresponding author.
