# Supplementary material for: Osteo‐F, a Newly Developed Herbal Formula, Ameliorates Osteoarthritis Through the NF‐κB/IκB/JNK Pathway Based on Network Pharmacology
Source: Food Sci Nutr. 2025 May 11;13(5):e70239. doi: 10.1002/fsn3.70239 (PMC12066244; doi:10.1002/fsn3.70239)
Supplement: Supplementary file 1 — Appendix S1 [file FSN3-13-e70239-s002.docx]

**Appendix S1. Supplementary data**
The following is the supplementary data related to this article: Supplementary data. 1. Osteo-F (*Schisandra chinensis* (Turcz.) Baill*, Lycium chinense* Mill.*, radix of Eucommia ulmoides* Oliv. in TM-MC database supplementary; 2. compounds and their related genes of Osteo-F; 3. Related genes of ingredient; 4. Related genes of Osteoarthritis (by disgenet); 5. Common genes of Osteo-F and Osteoarthritis; 6. Functional enrichment analysis (Common genes of Osteo-F and Osteoarthritis).
